# Lorlatinib and compound mutations in ALK+ large-cell neuroendocrine lung carcinoma: a case report

**DOI:** 10.1101/mcs.a006234

**Published:** 2022-10

**Authors:** Christiane Wiedemann, Daniel Kazdal, Jelena Cvetkovic, Julia Kunz, David Fisch, Martina Kirchner, Mark Kriegsmann, Holger Sültmann, Claus-Peter Heussel, Helge Bischoff, Michael Thomas, Albrecht Stenzinger, Petros Christopoulos

**Affiliations:** 1 Department of Thoracic Oncology, Thoraxklinik at Heidelberg University Hospital and National Center for Tumor Diseases, Heidelberg, 69126 Germany;; 2 Institute of Pathology, Heidelberg University Hospital, Heidelberg, 69120 Germany;; 3 Translational Lung Research Center Heidelberg (TLRC-H), member of the German Center for Lung Research (DZL), Heidelberg, 69120 Germany;; 4 Division of Cancer Genome Research, German Cancer Research Center (DKFZ), Heidelberg, 69120 Germany;; 5 Department of Radiology with Nuclear Medicine, Thoraxklinik at Heidelberg University Hospital, Heidelberg, 69126 Germany

**Keywords:** lung adenocarcinoma

## Abstract

Large-cell neuroendocrine lung carcinoma (LCNEC) is a high-grade neoplasm with median survival of 1 year and limited therapeutic options. Here, we report the unusual case of a 47-yr-old female smoker with stage IV LCNEC featuring *EML4*–*ALK* variant 2 (E20:A20), wild-type *TP53/RB1*, and low tumor mutational burden of 3.91 mut/Mb. Despite early progression within 3 mo under crizotinib, a durable response was achieved with alectinib. Oligoprogression in the left breast 10 mo later was treated by surgery, followed by a switch to ceritinib upon multifocal progression and detection of *ALK*:p.V1180L in the mastectomy specimen, but without success. Another rebiopsy revealed *ALK*:p.L1196M, but the tumor did not respond to brigatinib or carboplatin/pemetrexed, before stabilization under lorlatinib. Diffuse progression 8 mo later with detection of *ALK:*p.L1196M/p.G1202R and p.L1196M/ p.D1203N evolving from the previous p.L1196M did not respond to chemoimmunotherapy, and the patient succumbed with an overall survival (OS) of 37 mo. This case illustrates the importance of molecular profiling for LCNEC regardless of smoking status, and the superiority of next-generation ALK inhibitors compared to crizotinib for ALK+ cases. Lorlatinib retained efficacy in the heavily pretreated setting, whereas its upfront use could possibly have prevented the stepwise emergence of compound *ALK* mutations. Furthermore, the disease course was more aggressive and OS shorter compared to the V2/*TP53*wt ALK+ lung adenocarcinoma, whereas crizotinib, ceritinib, and brigatinib did not confer the benefit expected according to next-generation sequencing results, which also underline the need for more potent drugs against ALK in the high-risk setting of neuroendocrine histology.

## INTRODUCTION

Large-cell neuroendocrine lung carcinomas (LCNECs) are rare, high-grade neoplasms with poor survival and very limited therapeutic options (Lo Russo et al. 2016). They represent ∼3% of non-small-cell lung cancers (NSCLCs), but bear important biologic and clinical similarities with small-cell lung cancers (SCLCs), as well (Asamura et al. 2006): According to a real-world study, early-stage (I-III) LCNEC behaves like NSCLC, whereas metastatic LCNEC is more similar to SCLC (Kinslow et al. 2020).

Anaplastic lymphoma kinase (*ALK*) fusions drive ∼5% of lung adenocarcinomas (Williams et al. 2016). These tumors are a model disease among NSCLC because of their very low tumor mutational burden, <3 mut/Mb, longest patient survival, currently >5 yr in median, and emerging molecular risk stratification based on the *ALK* fusion variant and *TP53* status (Christopoulos et al. 2019b). Since 2007, when echinoderm microtubule-associated protein-like 4 (*EML4*)-*ALK* fusions were first described in lung cancer (Soda et al. 2007), several ALK tyrosine kinase inhibitors (TKIs) spanning three generations have been developed and are already approved for routine use in Europe: crizotinib (first generation); ceritinib, alectinib, and brigatinib (all second generation); and lorlatinib (third generation) (Elsayed and Christopoulos 2021). However, oncogenic driver mutations are exceedingly rare in LCNEC, and median survival does not exceed 12 mo under chemotherapy for these patients (Yamazaki et al. 2005; Sun et al. 2012).

## RESULTS

### Case Presentation

**Figure 1.**
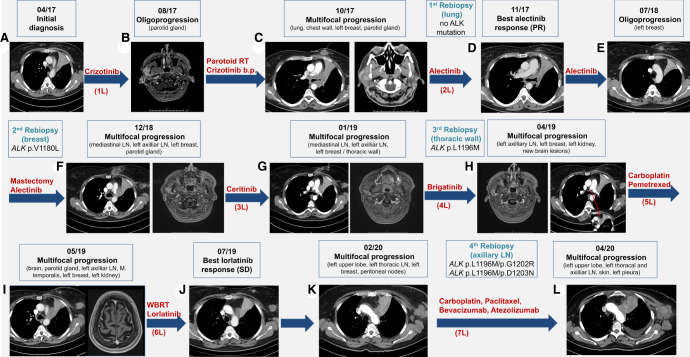
Radiologic findings over the course of the disease. (*A*) Chest computed tomography (CT) scan at initial diagnosis. (*B*) Head magnetic resonance imaging (MRI) 4 mo after crizotinib start with oligoprogression in the right parotid gland. (*C*) Chest and cranial CT scans with multifocal progression after radiotherapy. (*D*) Chest CT scan showing response to alectinib. (*E*) Chest CT scan showing oligoprogression in the left breast 8 mo later. (*F*) Chest CT and head MRI showing multifocal progression after partial mastectomy. (*G*,*H*) Chest CT and head MRI showing multifocal progression under certinib and brigatinib. (*I*) Chest CT and head MRI showing multifocal progression under carboplatin-pemetrexed. (*J*) Chest CT showing response to lorlatinib. (*K*) Chest CT and head MRI showing multifocal progression under lorlatinib. (*L*) Chest CT showing multifocal progression under carboplatin-paclitaxel-bevacizumab-atezolizumab; the patient died 3 wk later. (L) Treatment line, (RT) radiotherapy, (PR) partial response, (SD) stable disease, (LN) lymph nodes.

**Figure 2.**
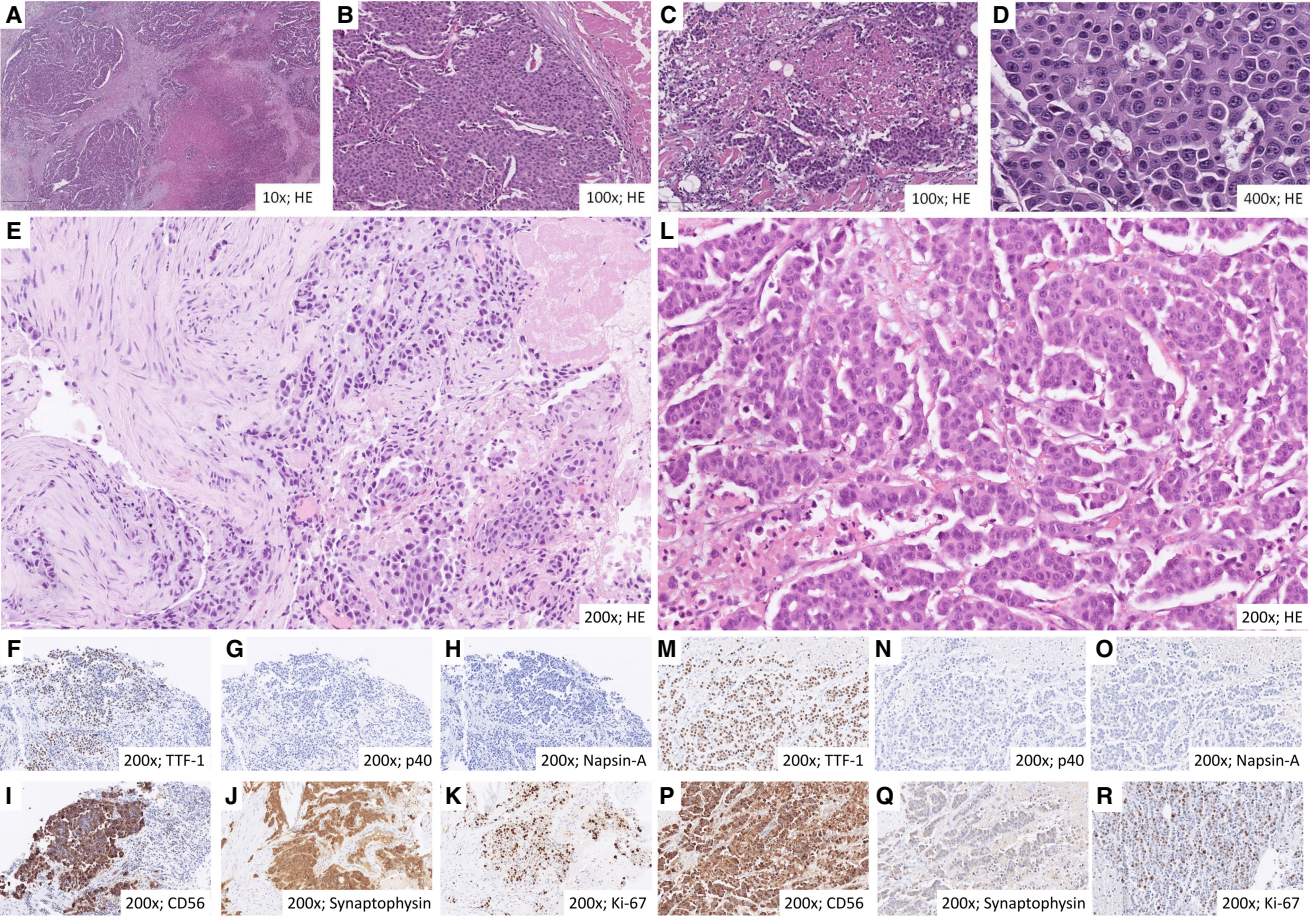
Histological characteristics of large-cell neuroendocrine lung carcinoma (LCNEC) in the initial biopsy of the lung tumor (*A*–*K*) and third tumor rebiopsy from a chest wall lesion in 02/2019 after ceritinib (*L*–*R*, see Table 1; Fig. 1). At initial diagnosis, the neoplasia showed a solid and trabecular growth pattern (*A*,*B*) with recognizable areal necrosis (*A*,*C*). The cells were medium- to large-sized, featured enlarged nuclei, granular karyoplasm with clearly recognizable nucleoli, and broad cytoplasm (*D*). Immunohistochemical staining was positive for TTF-1, CD56, and synaptophysin and negative for p40 and Napsin-A, and the Ki-67 proliferation rate was 40% (*E*–*K*). At the third tumor rebiopsy, tumor cells showed higher-grade features, including larger nuclei and more prominent nucleoli (*L*–*R*).

We report the unusual case of a 47-yr-old smoker female patient with a 20 pack per year tobacco history diagnosed with LCNEC of the left upper lung lobe in April 2017 during workup for intractable cough. Computed tomography (CT) of the chest/abdomen and brain magnetic resonance imaging (MRI) revealed metastatic lesions to multiple mediastinal lymph nodes, the liver, and the right adrenal gland, as well as three small (up to 5 mm) asymptomatic supratentorial lesions in the right cerebral hemisphere (Fig. 1A). A bronchoscopic biopsy of the lung tumor revealed an autochthonous CEA-, CK7-, calcitonin- and TTF1-positive neoplasm comprised of medium to large-sized cells with large nuclei, granular karyoplasm, clearly recognizable nucleoli, broad cytoplasm, solid and trabecular growth pattern, as well as focal necrosis (Fig. 2). Ultrasound examination of the neck revealed two suspicious cervical lymph nodes on the right side with a short-axis diameter up to 1.3 cm, but no thyroid gland lesion. Furthermore, the tumor cells strongly expressed CD56 and synaptophysin and showed a Ki-67 proliferation rate of 40%. The estrogen receptor was positive in 40%. PD-L1 showed no expression in tumor cells (tumor proportion score: 0) and was positive in single immune cells only (immune cell score: <1%). GATA3, progesterone receptor, HER2, cytokeratin-5/6, p40, p63, PAX8, SOX-10, and Napsin-A were all negative.

### Treatment

**Figure 3.**
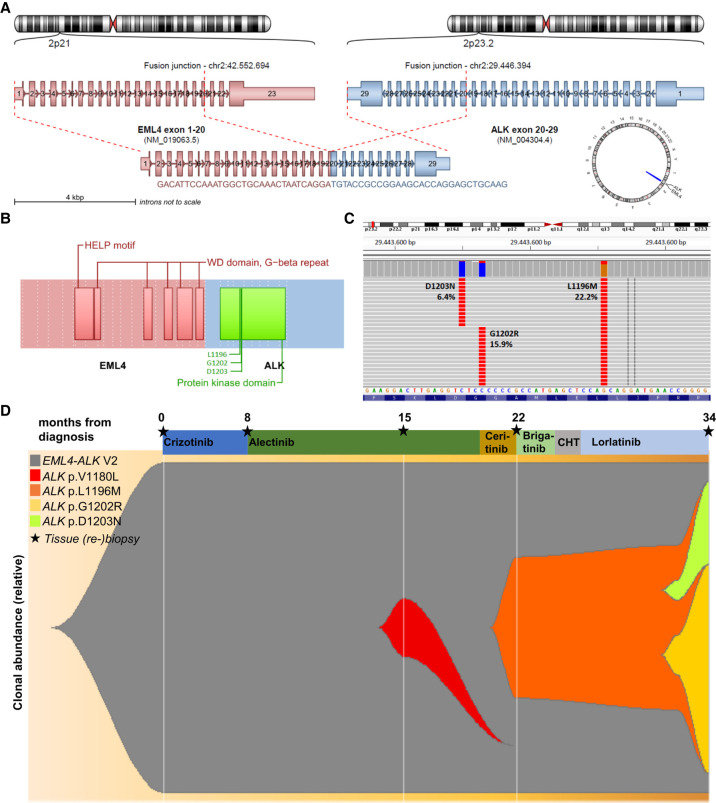
The primary gene fusion and secondary *ALK* mutations identified in the patient. (*A*) *EML4* exons 1–20 and *ALK* exons 20–29 were involved in the primary gene fusion (E20:A20, aka *EML4*–*ALK* variant 2). (*B*) The resulting chimeric protein with its main domains and the position of detected *ALK* resistance mutations in the last rebiopsy of the patient at the time of lorlatinib failure in 03/2020. (*C*) Reads of resistance mutations in the last rebiopsy of the patient at the time of lorlatinib failure in 03/2020, which reveals outgrowth of two distinct clones with compound mutations: p.L1196M in combination with p.D1203N, and p.L1196M in combination with *ALK* p.G1202R. Both have likely arisen on the ground of the preexistent p.L1196M mutation (Table 1; Fig. 1) because of the selective pressure under lorlatinib and are known to be highly lorlatinib resistant, as explained in the main text. (*D*) Fishplot of relative clonal abundance based on the genetic alterations detected in the patient's samples after correction for the tumor cell content (Table 1).

First-line crizotinib was initiated in Mai 2017 (Fig. 1) with stable disease (SD) as best response and early progression at the right parotid gland already after 3 mo, which was treated with radiotherapy. Upon multifocal progression in the left lung, chest wall, left breast, and left parotid gland 2 mo later, therapy was empirically switched to alectinib, which resulted in durable tumor shrinkage of >30%, corresponding to a partial response (PR) by RECIST v1.1. The treatment was generally well-tolerated without significant hemolysis, and a transient reduction of hemoglobin from 13.3 g/dL at baseline to 12.4 g/dL 1 wk later, gradually recovered within the next few weeks (Kunz et al. 2022). A second oligoprogression in the left breast 10 mo later was treated by surgical excision of the growing lesion, whose molecular workup revealed an *ALK*:p.V1180L mutation. Consequently, upon multifocal disease progression 3 mo later, alectinib was switched to ceritinib, but no tumor response was noted. Histological and molecular analysis of a growing chest wall lesion revealed *ALK*:p.L1196M. Consequently, the patient was switched to brigatinib, but showed further tumor growth under this drug, as well as under the subsequently administered carboplatin-pemetrexed chemotherapy, before achieving disease stabilization with lorlatinib, which lasted for ∼8 mo. In February 2020, tumor growth was noted again at several sites, along with detection of *ALK*:p.L1196M/p.G1202R and p.L1196M/p.D1203N compound mutations (Fig. 3C). Chemoimmunotherapy with carboplatin-paclitaxel-bevacizumab-atezolizumab showed no benefit, and the patient died 3 wk later in the palliative care unit. Overall survival (OS) was 37 mo.

### Molecular Findings

At the time of initial diagnosis in April 2017, molecular analysis by combined DNA and RNA next-generation sequencing (NGS) revealed the presence of an oncogenic gene fusion between *EML4* exons 1–20 and *ALK* exons 20–29 (E20:A20, aka *EML4*–*ALK* variant 2; Fig. 3A,B), but wild-type (wt) *TP53* and *RB1* (Table 1). A lung tissue rebiopsy analyzed with RNA NGS from an endobronchial rebiopsy at the time of crizotinib failure 6 mo later confirmed continued presence of the *ALK* fusion; therefore, alectinib was started. Analysis of a second tissue rebiopsy from the oligoprogressive breast lesion after excision in July 2018, at the time of alectinib failure, revealed an *ALK*:p.V1180L mutation, which according to in vitro data confers resistance to both crizotinib and alectinib, but is sensitive to ceritinib (Katayama et al. 2014). Analysis of a third tissue rebiopsy from a chest wall lesion at the time of ceritinib failure in February 2019 showed an *ALK*:p.L1196M mutation, which according to in vitro data confers resistance to crizotinib and alectinib, but is sensitive to brigatinib (Katayama et al. 2011; Gainor et al. 2016). In addition, a newly acquired *CDKN2A* deletion was detected at that time (Table 1), which is known to promote lung cancer progression, and the tumor cells showed a higher-grade morphology at this time compared to initial diagnosis (Fig. 2E–K vs. L–R) (Liu et al. 2020). Analysis of a fourth tissue rebiopsy from a growing axillary lymph node at the time of lorlatinib failure in March 2020 revealed the compound *ALK* mutations p.L1196M/p.G1202R and p.L1196M/p.D1203N, which are known to cause strong resistance against all approved ALK TKI, including lorlatinib (Yoda et al. 2018). The compound nature of these mutations was confirmed by examination of individual sequencing reads, which showed coexistence of *ALK*:p.L1196M with either *ALK*:p.D1203N, or *ALK*:p.G1202R (Fig. 3C). Given the previous detection of isolated *ALK*:p.L1196M mutation in February 2019, both compound mutations presumably arose in a preexisting *ALK*:p.L1196M-positive clone due to the selective pressure under lorlatinib (Fig. 3D; Yoda et al. 2018). All five analyzed samples of the patient showed the *EML4–ALK* V2 fusion, while there was no evidence of any other fusion or single-nucleotide variant (SNV) in the regions covered by our 38–42-gene panel (Table 1); in particular, *TP53*wt and *RB1*wt were confirmed by analysis of the second tumor rebiopsy using the TruSight Oncology 500 (TSO500) panel, which covers all *TP53* and *RB1* exons, including splice sites, as well as sensitive detection of copy-number variations, and showed no mutation or deletion of these two genes (Table 1). The TSO500 analysis also revealed a low tumor mutational burden of 3.91 mut/Mb (Table 1). The results and further details of sequencing experiments including ClinVar accession numbers for the detected variants are provided in Table 1 and Table 2.

**Table 1.** Results of DNA and RNA NGS in the five samples of the patient with our 38/42 gene panel

Date	mo	TCC	Fusion	SNV	CNV	Other
04/2017 (baseline)	0	20%	E20:A20^a^	None	None	None
10/2017 (first rebiopsy)	7	60%	E20:A20^a^	n/a (only RNA NGS, but no DNA NGS performed on this sample)		
07/2018 (second rebiopsy)	15	90%	E20:A20^a^	*ALK*:p.V1180L (VAF 8%)^b^	None	TMB 3.91 mut/Mb^b^
02/2019 (third rebiopsy)	22	80%	E20:A20^a^	*ALK*:p.L1196M (VAF 17.2%)	*CDKN2A*del	None
03/2020 (fourth rebiopsy)	34	50%	E20:A20^a^	*ALK*:p.L1196M (VAF 22.2%) *ALK*:p.D1203N (VAF 6.4%) *ALK*:p.G1202R (VAF 15.9%)	None	None

^a^*EML4-ALK* E20:A20 (V2).

^b^Analysis with the TSO500 panel showed the same *ALK*:p.V1180L SNV, as well as *PALB2*:p.E27* (ClinVar accession number SCV002571108), but no mutation or deletion in *TP53* and *RB1* (all exons and splice sites of both genes covered); the TMB was 3.91 mut/Mb.

(NGS) Next-generation sequencing, (mo) months after diagnosis, (TCC) tumor cell content, (SNV) single-nucleotide variants, (VAF) variant allele frequency, (CNV) copy-number variants, (TMB) tumor mutational burden.

**Table 2.** Details of the sequencing experiments and ClinVar accession numbers for the single-nucleotide variants of Table 1

Sequencing run					Detected variants									
Year	Mapped reads	Mean depth	Uniformity	On target	Accession number	Position	Muta-tion	Protein	cDNA	RefSeqID	Cove-rage	FW-Alt	RV-Alt	VAF
2017	365954	1593	96.44%	98.43%	No mutations detected									
2018	959431	3255	90.99%	97.28%	SCV002571104	Chr 2:29443679	C > G	*ALK*:p.Val1180Leu	c.3538G > C	NM_004304.5	2449	79	126	8.37
2019	785932	2406	91.10%	91.31%	SCV002571105	Chr 2:29443631	G > T	*ALK*:p.Leu1196Met	c.3586C > A	NM_004304.5	956	66	98	17.15
2020	987978	3296	83.70%	96.92%	SCV002571105	Chr 2:29443631	G > T	*ALK*:p.Leu1196Met	c.3586C > A	NM_004304.5	1111	93	154	22.19
					SCV002571107	Chr 2:29443613	C > T	*ALK*:p.Gly1202Arg	c.3604G > A	NM_004304.5	1105	67	105	15.91
					SCV002571106	Chr 2:29443610	C > T	*ALK*:p.Asp1203Asn	c.3607G > A	NM_004304.5	1106	26	45	6.43

(VAF) Variant allele frequency, (FW-Alt) number of forward reads with the mutation, (RV-Alt) number of reverse reads with the mutation.

## DISCUSSION

Comprehensive genetic studies have determined a very low frequency of actionable mutations in advanced LCNEC—for example, <2% for *ALK* fusions in two large series: 0/43 patients (Rekhtman et al. 2016) and 1/61 patients (Akhoundova et al. 2022). Their presence is of major clinical interest because of the poor prognosis and limited therapeutic options in this rare disease, so that eight patients with ALK+ LCNEC have been published so far, which are summarized in Table 3. Seven of them (7/8; Table 3) were never smokers; therefore, ALK+ LCNEC in an active smoker with 20 pack–years, as reported here, presumably represents an exceedingly rare constellation, <0.5% of LCNEC, and the second such published case, besides the 73-yr-old male smoker #8 of Table 3 with 30 pack-years. Thus, one first takeaway is that molecular workup can deliver clinically relevant results in LCNEC regardless of the patients’ age, sex and smoking status.

**Table 3.** Published cases of patients with stage IV ALK+ LCNEC (pure histologies)

Case #	Age/sex	Smoking	TKI	Response	Progression	OS	Reference
1	75/F	N	Alectinib	PR at 4 mo	n.r.	n.r.	Hayashi et al. 2018
2	37/M	N	Alectinib	PR at 4 wk	PD at 4 mo	≈9 mo	Leblanc et al. 2021
			Brigatinib	None	PD at 2 wk		
			Lorlatinib	None	PD at 4 wk		
3	43/F	N	Crizotinib	None	PD at 6 wk	n.r.	Omachi et al. 2014
4	51/M	N	Crizotinib	PR at 4 mo	PD at 10 mo	n.r.	Wang et al. 2019
5	37/M	N	Alectinib	PR at 4 wk	PD at 10 mo	>20 mo	Akhoundova et al. 2022
			Lorlatinib	SD ongoing			
6	32/F	N	Alectinib	PR at 6 wk	Ongoing	>6 mo	Akhoundova et al. 2022
7	68/F	N	Alectinib	None	PD at 1 wk	≈8 mo	Akhoundova et al. 2022
8	73/M	Y^a^	Crizotinib	SD	PD at 10 mo	>25 mo	Shimizu et al. 2018
			Alectinib	PR at 4 mo	Ongoing		
9	47/F	Y^b^	Crizotinib	SD	PD at 3 mo	37 mo	Current case
			Alectinib	PR at 2 mo	PD at 10 mo		
			Ceritinib	None	PD at 4 wk		
			Brigatinib	None	PD at 4 wk		
			Lorlatinib	SD	PD at 8 mo		

The current case is case #9 in this table.

^a^Active smoker, 30 pack-years.

^b^Active smoker, 20 pack-years.

(ALK) Anaplastic lymphoma kinase, (LCNEC) large-cell neuroendocrine lung carcinomas, (F) female, (M) male, (PR) partial remission, (SD) stable disease, (PD) progressive disease, (OS) overall survival, (mo) months, (wk) weeks.

Another interesting observation is that benefit from ALK inhibitors can vary widely across ALK+ LCNEC patients and compounds. Although responses to crizotinib have been reported (e.g., #4 in Table 3), there are also primary refractory cases (e.g., #3 in Table 3), while newer, next-generation ALK inhibitors appear to elicit stronger responses: For example, our patient showed a durable, 10-mo-long PR to second-line alectinib after early progression within 3 mo under first-line crizotinib, and also achieved an 8 mo-long disease stabilization under lorlatinib in the sixth therapy line (Table 3; Fig. 1). Of note, the progression-free survival (PFS) of 3 mo and treatment duration of 5 mo observed under crizotinib in our patient were considerably shorter than the median PFS of 10–12 mo for upfront use of this drug in phase three studies (Mok et al. 2020; Solomon et al. 2022), whereas the PFS of 10 and 8 mo observed in our patient under subsequent alectinib and lorlatinib, respectively, were not shorter (actually slightly longer) than the 7.1 and 6.6 mo reported for these drugs by phase 2 trials in the pretreated setting (Novello et al. 2018; Felip et al. 2021). It is also noteworthy that resistance to lorlatinib was caused by compound *ALK* mutations, whose development was facilitated by alectinib, under which the *ALK*:p.L1196M could emerge (Figs 1H and 3C,D). This stepwise development of ALK-dependent resistance under alectinib may be an argument for earlier use of lorlatinib in ALK+ LCNEC, besides the higher potency of the drug, as reflected in the limited clinical benefit from crizotinib, ceritinib, and brigatinib in our patient. Similar observations have been made in ALK+ lung adenocarcinoma: Also here, the main resistance mechanisms to next-line lorlatinib are compound *ALK* mutations, which affect ∼30% of patients (Shiba-Ishii et al. 2022) and are facilitated by previous treatment with second-generation drugs (Yoda et al. 2018). Of note, another published LCNEC case (#2 in Table 3) shows that lorlatinib may prove completely ineffective if given in later lines, even for patients who were initially TKI-sensitive.

Also remarkable is the aggressive clinical course of our patient, with several atypical metastatic sites (e.g., in the left breast and left parotid gland) within the first 6 mo. Such an early dissemination is typical for the presence of *EML4*–*ALK* v3 (E6;A20) and/or *TP53* mutations, whereas *EML4*–*ALK* v2 (E20;A20) *TP53*wt adenocarcinomas show a more indolent course and have a longer OS (Christopoulos et al. 2019a). Therefore, this ALK+ LCNEC patient demonstrates the adverse impact of LCNEC histology and how this can interact with molecular properties to shape an intermediate OS of 37 mo (Fig. 1) versus >5 yr in median for ALK+ adenocarcinomas, and 12 mo in median for other LCNEC (Mok et al. 2020; Fisch et al. 2021). Similar observations have also been made for EGFR+ lung cancer, which shows a shorter survival in case of squamous compared to adenocarcinoma histology (Christopoulos et al. 2020). In keeping with this, the tumor mutational burden in our ALK+ LCNEC case was 3.91 mut/Mb, which is significantly lower than that of other NSCLC-like LCNEC (average >10 mut/Mb) (Rekhtman et al. 2016), but higher than that of most ALK+ adenocarcinomas (mean < 3.0 mut/Mb, and even lower, <2 mut/Mb, for *TP53*wt cases) (Christopoulos et al. 2019b). Thus, ALK+ LCNEC emerges as a high-risk subset of ALK+ disease. Along the same lines, efficacy of ALK inhibitors did not correlate well with NGS results in our patient, as the tumor did not respond to ceritinib and brigatinib in the third and fourth lines, despite the presence of *ALK*:p.V1180L and *ALK*:p.L1196M, respectively, which are per se sensitive to these drugs (Fig. 1; Gainor et al. 2016). One molecular mechanism possibly related to rapid tumor growth and the lack of response to brigatinib was the *CDKN2A* deletion detected in the third tumor rebiopsy (Table 1), which promotes lung cancer progression and also impairs TKI responses in the similar constellation of *EGFR*-mutated lung cancer (Jiang et al. 2016; Liu et al. 2020).

Nonetheless, our index patient had an OS of 37 mo, which is longer than that of most published TKI-treated ALK+ LCNEC (Table 3). One main reason might be the administration of multiple ALK inhibitor and chemotherapy lines, because more different types and lines of treatment have been linked to longer survival in both ALK+NSCLC and LCNEC (Elsayed et al. 2021; Fisch et al. 2021). Of note, pemetrexed (fifth line) and paclitaxel (seventh line) were prioritized here as platinum partners over etoposide, based on the *TP53*wt and *RB1*wt status of the tumor, which suggest that the tumor may have an “NSCLC-like” biology (Rekhtman et al. 2016) with a possibly higher sensitivity to these cytotoxic drugs (Derks et al. 2018). A second reason for the relatively long OS might be repeat implementation of local therapies for oligoprogression (Fig. 1B,E), which confer additional survival benefit, as documented for both ALK+ and wild-type NSCLC (Weickhardt et al. 2012; Rheinheimer et al. 2020). Emerging strategies to further improve outcome of ALK+ LCNEC could be the upfront use of more potent, next-generation ALK inhibitors instead of crizotinib (Mok et al. 2020; Camidge et al. 2021; Solomon et al. 2022); closer patient surveillance using serial liquid biopsies to detect disease progression earlier (Angeles et al. 2021), monitor acquired resistance (McCoach et al. 2018; Dietz et al. 2020), and inform the choice between local ablation versus systemic therapy switch in case of oligoprogression (Christopoulos et al. 2021); use of PD-(L)1 inhibitors, which are associated with longer OS compared to chemotherapy in several retrospective analyses of LCNEC (Dudnik et al. 2021; Fisch et al. 2021); and implementation of novel, experimental compounds, such as the upcoming fourth-generation ALK TKI and drugs directed against other signaling pathways (Christopoulos et al. 2017; Ou et al. 2021; Andrini et al. 2022). At the same time, it should be noted that many of these approaches remain experimental without proven survival benefit and warrant systematic examination in prospective studies before wide adoption, as, for example, recently outlined by the U.S. Food and Drug Administration (FDA) in the guidance document on the integration of circulation tumor DNA (ctDNA) assays in clinical research (FDA 2022).

## CONCLUSION

In summary, this case underlines the potential clinical utility of broad genetic testing in patients with LCNEC, regardless of age, sex, and smoking status. Despite a more aggressive course than typical for ALK+ lung adenocarcinoma, our ALK+ LCNEC patient derived significant benefit from targeted and local therapies and achieved an OS of >3 yr. Earlier treatment with lorlatinib to prevent occurrence of compound mutations and use of other novel drugs could further improve prognosis of ALK+ LCNEC in the future.

## METHODS

Immunohistochemical staining was conducted using an autostainer (BenchMark ULTRA, Ventana Medical Systems) according to the manufacturer's instructions (please see Supplemental Table 1 for antibody details).

DNA was extracted automatically from six 10-µm formalin-fixed, paraffin-embedded (FFPE) sections of each sample by applying the Maxwell 16 FFPE Tissue LEV DNA Puriﬁcation Kit on a Maxwell 16 Research system (both Promega). DNA concentrations were determined with the Qubit HS DNA assay (Thermo Fisher Scientiﬁc) according to the manufacturers’ protocols.

A 38/42-gene custom DNA and RNA panel (Thermo Fisher Scientiﬁc) was used for targeted sequencing on the IonTorrent platform (AmpliSeq technology, Thermo Fisher Scientific) according to the manufacturers protocol as previously described (Volckmar et al. 2019). The covered genes and exons are given in Supplemental Tables 2 and 3. Mutation and CNV calling was completed by GATK 4.2. Fusion detection was performed from the RNA-seq panel as published (Volckmar et al. 2019). Clonal abundance was calculated by the variant allele frequencies of the detected mutations after correction for the tumor cell content (Table 1) and plotted using the fishplot R package (https://github.com/chrisamiller/fishplot) (Miller et al. 2016). Hybrid-capture based NGS with the TruSight Oncology 500 panel was performed on a NextSeq according to the manufacturer's instructions (Illumina).

## ADDITIONAL INFORMATION

### Data Deposition and Access

Mutations in Table 1 have been deposited in ClinVar (https://www.ncbi.nlm.nih.gov/clinvar/) with the accession numbers SCV002571104–SCV002571107, as shown in Table 2.

### Ethics Statement

Written consent was obtained from the patient included in this study. Ethical approval for the study was granted by the ethics committee of Heidelberg University (S-296/2016). The authors are accountable for all aspects of the work in ensuring that questions related to the accuracy or integrity of any part of the work are appropriately investigated and resolved. All procedures performed in studies involving human participants were in accordance with the ethical standards of the institutional and/or national research committee(s) and with the Helsinki Declaration (as revised in 2013).

### Author Contributions

C.W., J.K., D.K., and P.C. conceptualized the study. M.Ki., M.Kr., D.K., D.F., H.S., C.-P.H., A.S., and P.C. established the methodology. C.W., J.K., M.Kr., D.K., D.F., C.-P.H., and P.C. analyzed the data. C.W., D.F., C.-P.H., and P.C. interpreted the data. C.W., H.B., M.T., A.S., and P.C. provided resources. P.C., J.C., and C.W. wrote the original draft. All authors reviewed the writing and edited the manuscript. C.W., D.K., J.C., M.Kr., C.-P.H., and P.C. visualized the project. M.T., A.S., and P.C. supervised the project. All authors have approved the current version of the manuscript and its submission to *CSH Molecular Case Studies*.

### Funding

This research received funding from the German Center for Lung Research (DZL).

### Competing Interest Statement

C.W. reports speaker’s honoraria from GSK, Roche, and the German Society of Pneumology, as well as advisory board fees from Roche and educational fees from MSD and Boehringer Ingelheim. D.K. reports personal fees from AstraZeneca, Bristol-Myers Squibb, Pfizer, Lilly, Agilent, and Takeda outside the submitted work. A.S. reports advisory board honoraria from BMS, Astra Zeneca, Thermo Fisher, and Novartis; speaker’s honoraria from BMS, Illumina, Astra Zeneca, Novartis, Thermo Fisher, MSD, and Roche; and research funding from Chugai and BMS. M.T. reports advisory board honoraria from Novartis, Eli Lilly, BMS, MSD, Roche, Celgene, Takeda, AbbVie, Boehringer Ingelheim, and Pfizer; speaker’s honoraria from Eli Lilly, MSD, Takeda, and Pfizer; research funding from Astra Zeneca, BMS, Celgene, Novartis, Roche, and Takeda; and travel grants from BMS, MSD, Novartis, and Boehringer. P.C. reports research funding from Amgen, Astra Zeneca, Boehringer Ingelheim, Merck, Novartis, Roche, and Takeda; and advisory board/lecture fees from Astra Zeneca, Boehringer Ingelheim, Chugai, Daiichi Sankyo, Gilead, Novartis, Pfizer, Roche, and Takeda. All other authors have no conflict of interest to declare.

## Supplementary Material

Supplemental Material
