# Supplementary material for: Lorlatinib and compound mutations in ALK+ large-cell neuroendocrine lung carcinoma: a case report
Source: Cold Spring Harb Mol Case Stud. 2022 Oct;8(6):a006234. doi: 10.1101/mcs.a006234 (PMC9632356; doi:10.1101/mcs.a006234)
Supplement: Supplemental Material [file supp_8_6_a006234__DC1.html]

Supplemental Material 

# Lorlatinib and compound mutations in ALK+ large-cell neuroendocrine lung carcinoma: a case report

## Supplemental Material

- Supplemental\_Tables.docx
