# Supplementary material for: Lorlatinib and compound mutations in ALK+ large-cell neuroendocrine lung carcinoma: a case report
Source: Cold Spring Harb Mol Case Stud. 2022 Oct;8(6):a006234. doi: 10.1101/mcs.a006234 (PMC9632356; doi:10.1101/mcs.a006234)
Supplement: Supplemental Material [file supp_mcs.a006234_Supplemental_Tables.docx]

**SUPPLEMENTARY TABLE 1.** List of the antibodies used for immunohistochemistry in this study.

| **Antibody** | **Clone** | **Manufacturer** | **Dilution** |
| --- | --- | --- | --- |
| Calcitonin | SP17 | Cell Marque Corporation, Rocklin, CA, USA | Ready to use |
| CD56 | MRQ-42 | Cell Marque Corporation, Rocklin, CA, USA | Ready to use |
| CEA | CEA-31 | Cell Marque Corporation, Rocklin, CA, USA | Ready to use |
| CK5/6 | D5/16B4 | Ventana Medical Systems, Oro Valley, AZ, USA | Ready to use |
| CK7 | SP52 | Ventana Medical Systems, Oro Valley, AZ, USA | Ready to use |
| ER | SP1 | Ventana Medical Systems, Oro Valley, AZ, USA | Ready to use |
| GATA3 | L50-823 | Cell Marque Corporation, Rocklin, CA, USA | Ready to use |
| HER2 | SP3 | Cell Marque Corporation, Rocklin, CA, USA | 1:50 |
| Ki-67 | 30-9 | Ventana Medical Systems, Oro Valley, AZ, USA | Ready to use |
| Napsin-A | MRQ-60 | Cell Marque Corporation, Rocklin, CA, USA | Ready to use |
| p40 | BC28 | Ventana Medical Systems, Oro Valley, AZ, USA | Ready to use |
| p63 | 4A4 | Ventana Medical Systems, Oro Valley, AZ, USA | Ready to use |
| PAX8 | MRQ-50 | Cell Marque Corporation, Rocklin, CA, USA | Ready to use |
| PD-L1 | SP263 | Ventana Medical Systems, Oro Valley, AZ, USA | Ready to use |
| PR | PgR 1294 | Dako: Agilent, Santa Clara, CA, USA | 1:50 |
| SOX-10 | SP267 | Cell Marque Corporation, Rocklin, CA, USA | Ready to use |
| Synaptophysin | MRQ-40 | Cell Marque Corporation, Rocklin, CA, USA | Ready to use |
| TTF-1 | SPT24 | Leica Biosystems, Newcastle Upon Tyne, UK | 1:100 |
| CK 5/6 | D5/16B4 | Dako: Agilent, Santa Clara, CA, USA | 1:50 |
| TTF-1 | SPT24 | Leica Biosystems, Newcastle Upon Tyne, UK | 1:100 |
| CK7 | OV-TL 12/30 | Dako: Agilent, Santa Clara, CA, USA | 1:50 |
| p63 | SFI-6 | DCS Innovative Diagnostik-Systeme, Hamburg, DE | 1:100 |

**SUPPLEMENTARY TABLE 2.** Genes, exons and amplicons in our custom 38-42-gene DNA NGS panel. Please note that the second tumor rebiopsy was additionally analyzed using the TSO500 panel (Illumina, CA, USA), which covers all *TP53* and *RB1* exons and splice sites, and also offers sensitive detection of deletion for these and other genes (gene lists available online at the manufacturer’s website, https://emea.illumina.com/products/by-type/clinical-research-products/trusight-oncology-500.html).

| **Gene** | **Panel at baseline** | **Amp.** | **Panel used for the rebiopsies** | **Amp.** |
| --- | --- | --- | --- | --- |
| *ACVR2A* |  | 0 | 10 | 1 |
| *AKT* | 3 | 1 | 3 | 1 |
| *ALK* | 22, 23 | 2 | 22, 23, 24, 25 | 4 |
| *APC* |  | 0 | 16 | 1 |
| *ARID1A* | 2, 3, 10, 11, 14, 17*, 20 | 7 |  | 0 |
| *BRAF* | 11*, 15 | 2 | 11*, 15 | 2 |
| *CBL* | 2, 8, 9 | 3 |  | 0 |
| *CCND1* | 1, 3, 4 | 3 |  | 0 |
| *CCNE1* | 4*, 5, 10* | 3 |  | 0 |
| *CD274 (PD-L1)* |  | 0 | 3, 4 | 2 |
| *CDK6* | 3*, 5, 8 | 3 |  | 0 |
| *CDKN2A* | 1, 2* | 2 | 1, 2* | 2 |
| *CTNNB1* | 3*, 5 | 2 | 3 | 1 |
| *DDR2* | 5, 6, 7*, 8, 9, 11*, 12*, 13, 14, 15*, 16*, 17*, 18* | 13 |  | 0 |
| *EGFR* | 2*, 8*, 18*, 19*, 20*, 21* | 6 | all | 28 |
| *ERBB2 (HER2)* | 8*, 19*, 20 | 3 | 8, 17, 18, 19, 20, 21, 22 | 7 |
| *ERBB4 (HER4)* | 2*, 3, 6, 8*, 9*, 12, 15, 16*, 20, 21*, 22*, 23* | 12 | 2, 3, 6, 8, 9, 12, 15, 16, 21, 22, 23 | 10 |
| *EYS* | 5, 7, 9 | 3 |  | 0 |
| *FAM123B (AMER1)* | 1 | 1 |  | 0 |
| *FBXW7* | 9, 10*, 11 | 3 |  | 0 |
| *FGFR1* | 2*, 7, 10 | 3 | 2, 7, 10, 12, 13, 14 | 6 |
| *FGFR2* | 3, 7, 13 | 3 | 3, 7, 9, 12, 13, 14 | 6 |
| *FGFR3* | 7, 9, 16* | 3 | 7, 9, 14, 16 | 4 |
| *HRAS* | 2*, 3 | 2 |  | 0 |
| *JAK2* |  | 0 | 12, 13, 14 | 3 |
| *KEAP1* | 2, 3, 4, 5 | 4 | 2, 3, 4, 5 | 4 |
| *KIT* | 9*, 10*, 11* | 3 | 9, 10, 13, 17 | 4 |
| *KRAS* | 2*, 3*, 4* | 3 | 2, 3, 4 | 3 |
| *MAP2K1 (MEK1)* | 2 | 1 | 2, 3, 6 | 3 |
| *MCL-1* | 1, 3* | 2 |  | 0 |
| *MDM2* | 4*, 7, 11 | 3 |  | 0 |
| *MET* | 2, 14, 16*, 21 | 4 | 2, 8, 13, 14, 15, 16, 17, 18, 19, 20 | 9 |
| *MSH3* |  | 0 | 7 | 1 |
| *MYC* | 2, 3 | 2 |  | 0 |
| *NFE2L2* | 2* | 1 | 2* | 1 |
| *NKX2-1* |  | 0 | 1, 2 | 2 |
| *NOTCH1* |  | 0 |  | 0 |
| *NRAS* | 2*, 3, 4* | 3 | 2*, 3, 4* | 3 |
| *p14arf* |  | 0 | 1 | 1 |
| *PDGFRA* | 5*, 10, 11*, 12, 18*, 22 | 6 | 5, 10, 11, 12, 18, 22 | 6 |
| *PIK3CA* | 2, 5, 8*, 10*, 14, 21* | 6 | 2, 5, 8, 10, 14, 21 | 6 |
| *PIK3R1* |  | 0 | 3, 4, 5, 6, 7, 8 | 6 |
| *POLE* |  | 0 | 9, 14, 32, 33 | 4 |
| *PTEN* | 1, 2*, 3*, 5*, 6*, 7*, 8* | 7 | 1, 2, 3, 4, 5, 6, 7, 8, 9 | 9 |
| *RB1* | 2*, 3, 8*, 12*, 13*, 14*, 16*, 17, 18, 20, 21, 22*, 23 | 13 | 3, 8, 12, 13, 14, 16, 17, 18, 20, 21, 22, 23 | 12 |
| *RBM10* | 3, 6*, 18 | 3 |  | 0 |
| *RET* |  | 0 | 11, 12, 13, 14, 15, 16 | 6 |
| *RIT1* |  | 0 | 2, 3, 4, 5, 6 | 5 |
| *ROS1* |  | 0 | 2, 15, 36, 37, 38 | 5 |
| *SMAD4* | 5, 7*, 9, 12 | 4 |  | 0 |
| *SMARCA4* | 3*, 22*, 25, 26* | 4 |  | 0 |
| *SOX2* | 1 | 1 |  | 0 |
| *STK11* | 1, 2*, 4*, 6*, 8 | 5 | 1, 2, 4, 6, 8 | 5 |
| *TCF7L2* |  | 0 | 5, 14 | 2 |
| *TERT* | 2, 11 | 2 |  | 0 |
| *TP53* | 4, 5*, 6*, 7*, 8*, 9*, 10* | 7 | 4, 5, 6, 7, 8, 9, 10 | 7 |
| *U2AF1* |  | 0 | 2, 6 | 2 |
|  |  |  |  |  |
| Sum | 42 Genes | 164 | 38 Genes | 184 |

Amp.: Amplicon number; * marks exons fully covered by amplicons

**SUPPLEMENTARY TABLE 3.** Fusion partners detected by the RNA NGS panel used for detection of oncogenic fusions.

| **ALK** | **ROS1** | **RET** | **NTRK/other** |
| --- | --- | --- | --- |
| **ALK-PTPN3** | **CCDC6-ROS1** | **AFAP1-RET** | **AXL-MBIP** |
| **(A11P3)** | **(C5R35)** | **(A4R12)** | **(A20M4)** |
| **C2orf44-ALK** | **CD74-ROS1** | **AKAP13-RET** | **BAG4-FGFR1** |
| **(C4A20)** | **(C4R33)** | **(A36R12)** | **(B2F6)** |
| **CLIP4-ALK** | CD74-ROS1 | CCDC6-RET | **BRD4-NUTM1** |
| **(C12A23)** | (C6R32) | (C1R12) | **(B11N2)** |
| **CLTC-ALK** | CD74-ROS1 | **CCDC6-RET** | **CD74-NRG1** |
| **(C30A20)** | (C6R34) | **(C2R12)** | **(C6N6)** |
| EML4-ALK | **CEP85L-ROS1** | **CCDC6-RET** | **CD74-NRG1** |
| (E13A20) | **(C12R35)** | **(C8R11)** | **(C8N6)** |
| EML4-ALK | **CEP85L-ROS1** | CUX1-RET | CD74-NTRK1 |
| (E14A20) | **(C8R36)** | (C10R12) | (C8N12) |
| EML4-ALK | EZR-ROS1 | **ERC1_ELKS-RET** | CEL-NTRK1 |
| (E15A20) | (E10R34) | **(E11R12)** | (C7N7) |
| EML4-ALK | **GOPC-ROS1** | **ERC1-RET** | **CRTC1-MAML2** |
| (E17A20) | **(G4R35)** | **(E12R12)** | **(C1M2)** |
| EML4-ALK | GOPC-ROS1 | **ERC1-RET** | **EGFR-RAD51** |
| (E18A20) | (G4R36) | **(E17R12)** | **(E23R3)** |
| EML4-ALK | GOPC-ROS1 | **ERC1-RET** | **EZR-ERBB4** |
| (E20A20) | (G8R35) | **(E5R12)** | **(E11E18)** |
| EML4-ALK | LRIG3-ROS1 | **ERC1-RET** | **FGFR2-CIT** |
| (E2A20) | (L16R35) | **(E6R12)** | **(F17C23)** |
| **EML4-ALK** | SDC4-ROS1 | **ERC1-RET** | **FGFR2-IAA1967_CCAR2** |
| **(E6A17)** | (S2R32) | **(E7R12)** | **(F17C4)** |
| **EML4-ALK** | **SDC4-ROS1** | **FKBP15-RET** | **FGFR3-BAIAP2L1** |
| **(E6A18)** | **(S2R34)** | **(F25R12)** | **(F17B2)** |
| EML4-ALK | **SDC4-ROS1** | **GOLGA5_PTC5-RET** | **FGFR3-TACC3** |
| (E6A19) | **(S4R32)** | **(G7R12)** | **(F15T11)** |
| EML4-ALK | SDC4-ROS1 | **KIAA1468-RET** | **FGFR3-TACC3** |
| (E6A20) | (S4R34) | **(K10R12)** | **(F16T10)** |
| EML4-ALK | SLC34A2-ROS1 | KIF5B-RET | **FGFR3-TACC3** |
| (E6bA20) | (S13R32) | (K15R11) | **(F16T11)** |
| **EML4-ALK** | SLC34A2-ROS1 | KIF5B-RET | **FGFR3-TACC3** |
| **(E7A20)** | (S13R34) | (K15R12) | **(F17T10)** |
| **FN1-ALK** | **SLC34A2-ROS1** | KIF5B-RET | **FGFR3-TACC3** |
| **(F23A19)** | **(S13R36)** | (K16R12) | **(F17T13)** |
| HIP1-ALK | SLC34A2-ROS1 | KIF5B-RET | **FGFR3-TACC3** |
| (H21A20) | (S4R32) | (K22R12) | **(F17T4)** |
| HIP1-ALK | SLC34A2-ROS1 | KIF5B-RET | **FGFR3-TACC3** |
| (H28A20) | (S4R34) | (K23R12) | **(F17T5)** |
| **KIF5B-ALK** | **TFG-ROS1** | **KIF5B-RET** | **FGFR3-TACC3** |
| **(K15A19)** | **(T4R34)** | **(K24R11)** | **(F17T8)** |
| KIF5B-ALK | **TMEM106B-ROS1** | **KIF5B-RET** | **FGFR3-TACC3** |
| (K15A20) | **(T3R35)** | **(K24R7)** | **(F17T9)** |
| KIF5B-ALK | **TPD52L1-ROS1** | KIF5B-RET | **FGFR3-TACC3** |
| (K17A20) | **(T3R33)** | (K24R8) | **(F18T7)** |
| **KIF5B-ALK** | TPM3-ROS1 | **KTN1-RET** | **FGFR3-TACC3** |
| **(K24A20)** | (T8R35) | **(K29R12)** | **(F18T8)** |
| KLC1-ALK |  | **NCOA4-RET** | **MIR548F1-NTRK1** |
| (K9A20) |  | **(N6R12)** | **(MN10)** |
| **MSN-ALK** |  | **NCOA4-RET** | MPRIP-NTRK1 |
| **(M11A20)** |  | **(N7R12)** | (M14N12) |
| **MYH9-ALK** |  | **NCOA4-RET** | MPRIP-NTRK1 |
| **(M34A20)** |  | **(N8R11)** | (M18N12) |
| **NCOA1-ALK** |  | **NCOA4-RET** | MPRIP-NTRK1 |
| **(N21A1)** |  | **(N8R12)** | (M21N12) |
| **NPM1-ALK** |  | **PARG-RET** | **MPRIP-NTRK1** |
| **(N4A20)** |  | **(P18R12)** | **(M21N14)** |
| **NPM1-ALK** |  | **PCM1_PTC4-RET** | NFASC-NTRK1 |
| **(N5A20)** |  | **(P29R12)** | (N20N10) |
| **PPFIBP1-ALK** |  | **PCM1-RET** | **NTRK1-TFG** |
| **(P12A20)** |  | **(P31R12)** | **(N9T6)** |
| **PPFIBP1-ALK** |  | **PRKAR1A-RET** | **NTRK1-TPR** |
| **(P8A20)** |  | **(P7R12)** | **(N12T6)** |
| **PRKAR1A-ALK** |  | **PRKAR1A-RET** | **NTRK1-TPR** |
| **(P2A20)** |  | **(P8R12)** | **(N9T22)** |
| **RNF213-ALK** |  | **RET-NCOA4** | **SCAF11-PDGFRA** |
| **(R20A20)** |  | **(R11N8)** | **(S1P2)** |
| **SEC31A-ALK** |  | **RET-NCOA4** | **SLC3A2-NRG1** |
| **(S21A20)** |  | **(R11N9)** | **(S5N6)** |
| **SQSTM1-ALK** |  | **RET-TRIM33** | SQSTM1-NTRK1 |
| **(S5A20)** |  | **(R11T17)** | (S5N10) |
| **TFG-ALK** |  | **TBL1XR1-RET** | SSBP2-NTRK1 |
| **(T4A20)** |  | **(T9R11)** | (S12N12) |
| **TFG-ALK** |  | **TBL1XR1-RET** | **TCF3-PBX1** |
| **(T5A20)** |  | **(T9R12)** | **(T16P3)** |
| **TFG-ALK** |  | **TRIM24_PTC6-RET** | TFG-NTRK1 |
| **(T6A20)** |  | **(T9R12)** | (T5N10) |
| **TPM1-ALK** |  | **TRIM24-RET** | **TFG-NTRK1** |
| **(T8A20)** |  | **(T10R12)** | **(T5N9)** |
| **TPM3-ALK** |  | **TRIM24-RET** | **TFG-NTRK1** |
| **(T7A20)** |  | **(T9R12)** | **(T6N10)** |
| **TPM4-ALK** |  | **TRIM27-RET** | **TPM3-NTRK1** |
| **(T7A20)** |  | **(R6R10)** | **(T7N10)** |
| TPR-ALK |  | **TRIM27-RET** | **TPR-NTRK1** |
| (T15A20) |  | **(T3R12)** | **(T21N10)** |
| **VCL-ALK** |  | **TRIM33_PTC7-RET** | **TRIM24-BRAF** |
| **(V16A20)** |  | **(T16R12)** | **(T5B8)** |
|  |  | **TRIM33-RET** | **TRIM24-BRAF** |
|  |  | **(T14R12)** | **(T9B9)** |
|  |  |  | **VAMP2-NRG1** |
|  |  |  | **(V6N5)** |

Fused exons are given in parentheses
